# Characterization of the nucleolar localization signal of TRMT10A and its importance for the m^1^G9 methylation of tRNAs in mammalian cells

**DOI:** 10.1093/jmcb/mjaf011

**Published:** 2025-03-17

**Authors:** Tianyang Luo, Zhiyuan Shi, Haibin Yang, Jiafan Miao, Zilong Chang, Jie Zou, Qiang Zeng, Wenbin Wu, Yanan Jiang, Xiaoling Xie, Liu Cao, Hong Peng, Chunmei Li, Deyin Guo, Junyu Wu

**Affiliations:** Shenzhen Key Laboratory of Systems Medicine for Inflammatory Diseases, Centre for Infection and Immunity Study (CIIS), School of Medicine, Shenzhen Campus of Sun Yat-sen University, Shenzhen 518197, China; Shenzhen Key Laboratory of Systems Medicine for Inflammatory Diseases, Centre for Infection and Immunity Study (CIIS), School of Medicine, Shenzhen Campus of Sun Yat-sen University, Shenzhen 518197, China; Shenzhen Key Laboratory of Systems Medicine for Inflammatory Diseases, Centre for Infection and Immunity Study (CIIS), School of Medicine, Shenzhen Campus of Sun Yat-sen University, Shenzhen 518197, China; Shenzhen Key Laboratory of Systems Medicine for Inflammatory Diseases, Centre for Infection and Immunity Study (CIIS), School of Medicine, Shenzhen Campus of Sun Yat-sen University, Shenzhen 518197, China; Shenzhen Key Laboratory of Systems Medicine for Inflammatory Diseases, Centre for Infection and Immunity Study (CIIS), School of Medicine, Shenzhen Campus of Sun Yat-sen University, Shenzhen 518197, China; Shenzhen Key Laboratory of Systems Medicine for Inflammatory Diseases, Centre for Infection and Immunity Study (CIIS), School of Medicine, Shenzhen Campus of Sun Yat-sen University, Shenzhen 518197, China; Shenzhen Key Laboratory of Systems Medicine for Inflammatory Diseases, Centre for Infection and Immunity Study (CIIS), School of Medicine, Shenzhen Campus of Sun Yat-sen University, Shenzhen 518197, China; Shenzhen Key Laboratory of Systems Medicine for Inflammatory Diseases, Centre for Infection and Immunity Study (CIIS), School of Medicine, Shenzhen Campus of Sun Yat-sen University, Shenzhen 518197, China; Shenzhen Key Laboratory of Systems Medicine for Inflammatory Diseases, Centre for Infection and Immunity Study (CIIS), School of Medicine, Shenzhen Campus of Sun Yat-sen University, Shenzhen 518197, China; Shenzhen Key Laboratory of Systems Medicine for Inflammatory Diseases, Centre for Infection and Immunity Study (CIIS), School of Medicine, Shenzhen Campus of Sun Yat-sen University, Shenzhen 518197, China; Shenzhen Key Laboratory of Systems Medicine for Inflammatory Diseases, Centre for Infection and Immunity Study (CIIS), School of Medicine, Shenzhen Campus of Sun Yat-sen University, Shenzhen 518197, China; Shenzhen Key Laboratory of Systems Medicine for Inflammatory Diseases, Centre for Infection and Immunity Study (CIIS), School of Medicine, Shenzhen Campus of Sun Yat-sen University, Shenzhen 518197, China; Shenzhen Key Laboratory of Systems Medicine for Inflammatory Diseases, Centre for Infection and Immunity Study (CIIS), School of Medicine, Shenzhen Campus of Sun Yat-sen University, Shenzhen 518197, China; Guangzhou National Laboratory, Guangzhou International Bio-Island, Guangzhou 510320, China; MOE Key Laboratory of Tropical Disease Control, Institute of Human Virology, Department of Pathogen Biology and Biosecurity, Zhongshan School of Medicine, Sun Yat-sen University, Guangzhou 510080, China; State Key Laboratory of Respiratory Disease, National Clinical Research Center for Respiratory Disease, Guangzhou Institute of Respiratory Health, the First Affiliated Hospital of Guangzhou Medical University, Guangzhou 510182, China; Shenzhen Key Laboratory of Systems Medicine for Inflammatory Diseases, Centre for Infection and Immunity Study (CIIS), School of Medicine, Shenzhen Campus of Sun Yat-sen University, Shenzhen 518197, China

**Dear Editor**,

Transfer RNA (tRNA) is an indispensable adaptor molecule in the messenger RNA (mRNA) translation machinery, facilitating the conversion of genetic information encoded in mRNA into functional proteins. Numerous posttranscriptional modifications in tRNA have been identified, which play significant roles in modulating tRNA folding, biochemical stability, amino-acylation, and codon–anticodon interaction (Suzuki, 2021). TRMT10A, the mammalian homolog of Trm10, incorporates *N*^1^-methylguanosine modification at position 9 (m^1^G9) of various cytoplasmic tRNAs, including tRNA^Gln^ and tRNA^IniMeth^ (Vilardo et al., 2020). Mutations in human TRMT10A, which is enriched in pancreatic islets and brain (Igoillo-Esteve et al., 2013), are often associated with microcephaly, intellectual disability, early-onset diabetes, and short stature (Igoillo-Esteve et al., 2013; Uçan Tokuç et al., 2024). Although the predominant nucleolar localization of TRMT10A has been observed (Igoillo-Esteve et al., 2013), the molecular basis of the subcellular localization of TRMT10A remains unclear and whether the nucleolar localization contributes to its methyltransferase function is of great interest.

To confirm the subcellular localization of TRMT10A, we cloned the human TRMT10A gene into the pEGFP-N1 vector to create a fusion protein with green fluorescent protein (GFP). After transfected into 293T cells, the TRMT10A-GFP fusion protein was detected predominantly inside the nucleus and appeared to form large puncta (Figure 1A). Furthermore, immunofluorescence staining using nucleophosmin (NPM1) as the nucleolar marker verified colocalization of the TRMT10A puncta and the nucleolus in both HeLa and U251 cells (Figure 1B; Supplementary  Figure S1A). Consistently, the endogenous TRMT10A also preferentially accumulated inside the nucleolus in both HeLa and U251 cells (Figure 1C; Supplementary Figure S1B). These results indicate that both endogenous and GFP-tagged TRMT10A predominantly localize to the nucleolus.

**Figure 1:**
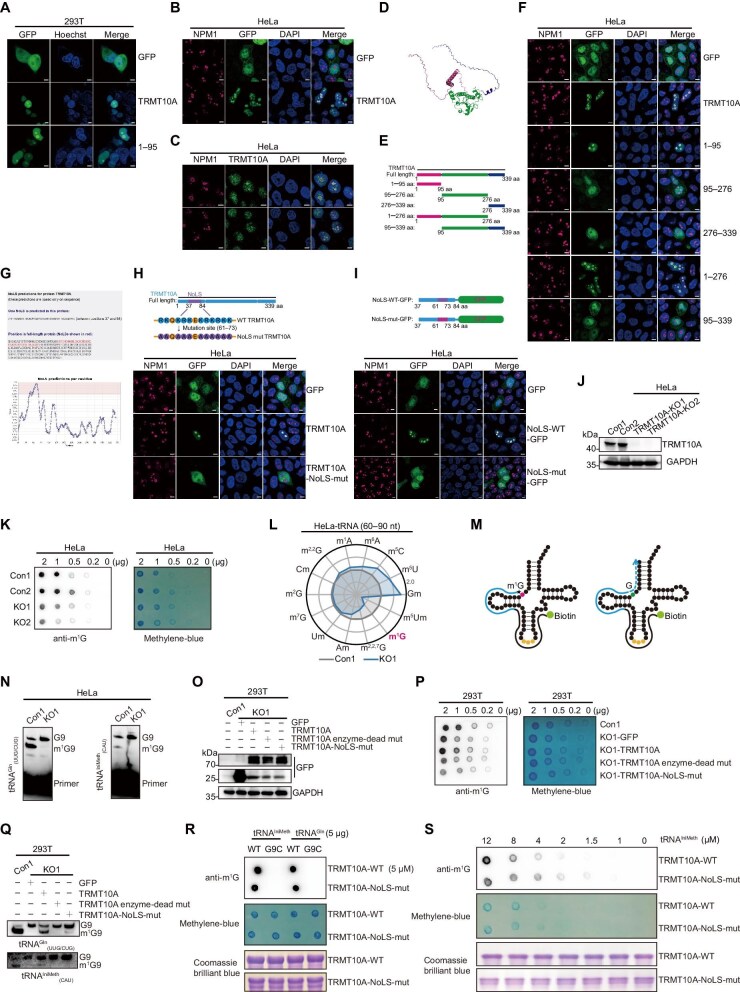
Nucleolar localization is critical for TRMT10A to modify m^1^G9 of its substrate tRNAs in cells. (**A**) The localization of TRMT10A and its N-terminal truncation in live 293T cells. The nuclei were visualized by staining with Hoechst33342. Scale bar, 5 μm. (**B**) The subcellular distribution of GFP-tagged TRMT10A in HeLa cells transfected with pEGFP-TRMT10A. NPM1 serves as a nucleolar marker. The nuclei were visualized by staining with DAPI. Scale bar, 5 μm. (**C**) Immunofluorescence staining of HeLa cells using anti-TRMT10A (green), anti-NPM1 (magenta), and DAPI (blue). Scale bar, 5 μm. (**D**) The three-dimensional structure of TRMT10A predicted by AlphaFold2. Magenta color represents the N-terminal domain, green color represents the methyltransferase domain, and blue color represents the C-terminal domain. (**E**) Schematic diagram showing the constructing strategy for TRMT10A truncations. (**F**) The subcellular distribution of GFP-tagged TRMT10A truncations in HeLa cells transfected with the indicated plasmids. Scale bar, 5 μm. (**G**) The aa37–84 (highlighted in magenta) was predicted to be the potential NoLS in TRMT10A by using the online NoD web tool. (**H**) The NoLS mutation blocks the nucleolus localization of TRMT10A. Upper panel: schematic diagram of the potential NoLS in TRMT10A and its mutant construction. Lower panel: the subcellular localizations of the indicated constructs in HeLa cells. Scale bar, 5 μm. (**I**) The NoLS of TRMT10A is sufficient to locate protein to the nucleolus. Upper panel: the NoLS of TRMT10A and its mutated sequence fused with GFP. Lower panel: the subcellular localizations of the indicated fusion proteins in HeLa cells. Scale bar, 5 μm. (**J**) Western blot analysis confirming the knockout efficiency of TRMT10A in HeLa cells. Two control cell lines and two TRMT10A-KO cell lines were used. GAPDH was used as a loading control. (**K**) Dot blot analysis to detect m^1^G modification levels of small RNAs (<200 nt) isolated from TRMT10A-KO and wild-type HeLa cells. The methylene blue staining was the loading control. (**L**) Quantification of RNA modifications in small RNAs (60–90 nt) isolated from TRMT10A-KO and wild-type HeLa cells by LC–MS/MS analysis. (**M**) Schematic diagram of the primer extension assay for monitoring m^1^G9 level in tRNA. The primer is indicated in black and the extension products are shown in blue. (**N**) Detection of m^1^G9 levels in tRNA^Gln^_(UUG/CUG)_ and tRNA^IniMeth^_(CAU)_ from TRMT10A-KO and wild-type HeLa cells using the primer extension assay. (**O**) Western blot analysis of protein levels of TRMT10A and its mutants overexpressed in TRMT10A-KO 293T cells. (**P**) Dot blot analysis to detect the levels of m^1^G modification in small RNAs (<200 nt) from TRMT10A-KO 293T cells transfected with the indicated plasmids. The methylene blue staining was the loading control. (**Q**) Detection of m^1^G9 levels in tRNA^Gln^_(UUG/CUG)_ and tRNA^IniMeth^_(CAU)_ from TRMT10A-KO 293T cells transfected with the indicated plasmids using the primer extension assay. (**R**) The methyltransferase activity of wild-type TRMT10A or the NoLS mutant to tRNA substrates (tRNA^IniMeth^, tRNA^IniMeth^-G9C, tRNA^Gln^, or tRNA^Gln^-G9C) was determined using anti-m^1^G dot blot analysis. The methylene blue staining and Coomassie Brilliant blue staining were the loading control. (**S**) The enzymatic kinetics of wild-type TRMT10A or the NoLS mutant for tRNA^IniMeth^ were determined with increasing concentrations of tRNA^IniMeth^ (0, 1, 1.5, 2, 4, 8, and 12 μM) and 1 μM enzyme.

We employed AlphaFold2 to predict the three-dimensional structure of TRMT10A (Figure 1D), which revealed that, in addition to the methyltransferase central region, TRMT10A contains disordered regions at both N- and C-termini. We then superimposed our predicted structure with the crystal structure of the TRMT10A methyltransferase domain (PDB 4FMW) using PyMOL software and found a good consistency between the two structures (Supplementary Figure S2A). Based on the predicted structure, several GFP-tagged TRMT10A truncations were constructed, including TRMT10A-aa1–95, TRMT10A-aa95–276, TRMT10A-aa276–339, TRMT10A-aa1–276, and TRMT10A-aa95–339 (Figure 1E). Western blot analysis confirmed the successful construction of these truncations with the correct molecular weights (Supplementary Figure S2B). Subsequently, the subcellular localizations of these truncations in HeLa and U251 cells were visualized via immunofluorescence. Interestingly, both TRMT10A-aa1–95 and TRMT10A-aa1–276 were concentrated in the nucleolus similar to the full-length protein, whereas TRMT10A-aa95–276, TRMT10A-aa276–339, and TRMT10A-aa95–339 were diffused in the cytoplasm and nucleoplasm (Figure 1F; Supplementary Figure S2C). In addition, the TRMT10A-aa1–95-GFP fusion protein formed large puncta, similar to the full-length protein, in the nucleus of live 293T cells (Figure 1A). Therefore, our results suggest that the aa1–95 region of TRMT10A is sufficient for the nucleolar localization of this protein.

By using a nucleolar localization prediction tool, the NoD web tool (http://www.compbio.dundee.ac.uk/www-nod) (Scott et al., 2011), aa37–84 of TRMT10A was identified as a putative nucleolar localization sequence (NoLS) (Figure 1G). This result further supports our conclusion that the aa1–95 region of TRMT10A is responsible for its nucleolar concentration. NoLS motifs often contain positively charged amino acid residues such as lysine (K), arginine (R), and histidine (H) (Martin et al., 2015). A highly positive-charged peptide ‘^61^RKQKRKEKRKRKK^73^’ was found within the predicted NoLS of TRMT10A (Figure 1H, upper panel). We then mutated all R and K residues within aa61–73 to alanine (A) to construct pEGFP-TRMT10A-NoLS-mut (Figure 1H, upper panel). After transfected into HeLa, U251, and liver 293T cells, different from wild-type TRMT10A, the NoLS mutant no longer colocalized with NPM1 (Figure 1H, lower panel; Supplementary Figure S3A–C). To further validate the predicted NoLS, the aa37–84 peptides of wild-type and mutant TRMT10A were directly fused with GFP protein, constructing NoLS-WT-GFP and NoLS-mut-GFP, respectively (Figure 1I, upper panel). Immunofluorescence results demonstrated that NoLS-WT-GFP localized to the nucleolus, while NoLS-mut-GFP diffused into the cytoplasm and nucleoplasm in HeLa, U251, and live 293T cells (Figure 1I, lower panel; Supplementary Figure S3D–F). Altogether, these findings indicate that the aa37–84 region is responsible for the nucleolar localization of TRMT10A.

TRMT10A specifically catalyzes the methylation at the G9 site of tRNAs. To examine whether the nucleolar localization of TRMT10A participates in the modification of m^1^G9 methylation in tRNAs, we first established TRMT10A-knockout (TRMT10A-KO) cell lines in HeLa and 293T cells using the CRISPR/Cas9 system. Western blot analysis confirmed the successful deletion of TRMT10A in both HeLa (Figure 1J) and 293T (Supplementary Figure S4A) cells. Dot blot analysis of small RNAs (<200 nt) showed apparent reduction of m^1^G levels in both TRMT10A-KO HeLa cells (Figure 1K) and 293T cells (Supplementary Figure S4B). The quantification of RNA modifications in total tRNA nucleosides by liquid chromatography with tandem mass spectrometry (LC–MS/MS) analysis indicated that the m^1^G modification level in TRMT10A-KO cells dropped to ~50% of that in control cells (Figure 1L). We speculate that the residual m^1^G modification may be from the tRNA m^1^G37 methylation, which is mediated by another methyltransferase TRMT5 (Zhou et al., 2018). Among various tRNA modifications, the Gm, m^5^U, m^5^C, and m^6^A levels increased in TRMT10A-KO cells, while the m^2,2,7^G level decreased in a less degree than the m^1^G level (Figure 1L). The interplay between m^1^G9 and these tRNA modifications was largely unknown, except that TRMT10A has been reported to interact with the demethylase FTO and negatively regulate the m^6^A level (Ontiveros et al., 2020). It would be interesting to investigate the crosstalk of different tRNA modifications in the future studies.

The primer extension assay was often used to assess the methylation modifications at a specific site of individual tRNA, relying on the steric hindrance to prevent Watson–Crick base pairing (Swinehart et al., 2013). As shown in Figure 1M, RNA modification would block the primer extension during reverse transcription, resulting in a shorter DNA product. We thus performed the primer extension assay to evaluate the m^1^G levels in tRNA^Gln^_(UUG/CUG)_ and tRNA^IniMeth^_(CAU)_, two reported substrates of TRMT10A (Vilardo et al., 2020). Two amplicons were visualized from both tRNA^Gln^_(UUG/CUG)_ and tRNA^IniMeth^_(CAU)_ in wild-type HeLa cells, indicating that these two tRNAs were partially modified at the G9 site (Figure 1N). The shorter amplicons were virtually absent in TRMT10A-KO HeLa cells (Figure 1N), implying the dramatical reduction of m^1^G9 level in these two tRNAs. Altogether, the detection systems to evaluate tRNA m^1^G9 level were successfully established and our results confirmed that TRMT10A is the methyltransferase to modify m^1^G9 in various tRNAs.

To investigate the effects of the nucleolar localization of TRMT10A on tRNA m^1^G9 modification, rescue experiments were performed by transfecting the related constructs into TRMT10A-KO 293T cells (Figure 1O). As a control, the TRMT10A enzyme-dead mutant (G206R, V209A, and D210A) was constructed by destroying its *S*-adenosyl methionine (SAM) binding motif (Krishnamohan and Jackman, 2017). The enzyme-dead mutation in TRMT10A did not affect its nucleolar localization (Supplementary Figure S5). As expected, the dot blot assay showed that wild-type TRMT10A apparently increased the m^1^G modification level in small RNAs, while the enzyme-dead mutant failed to rescue this phenotype (Figure 1P). More interestingly, the NoLS mutant of TRMT10A showed minimal effects on the m^1^G modification level in TRMT10A-KO 293T cells (Figure 1P). The primer extension assay also showed that only wild-type TRMT10A restored the m^1^G9 modification level in the tRNA^IniMeth^_(CAU)_ and tRNA^Gln^_(UUG/CUG)_, while very minimal signal of m^1^G9 modification, if any, appeared in these two substrates after expression of the NoLS mutant in TRMT10A-KO 293T cells (Figure 1Q). To further validate the effects of NoLS mutation to the enzyme activity, we *in vitro* transcribed tRNA substrates and purified the recombinant wild-type TRMT10A and the NoLS mutant (Supplementary Figure S6). The *in vitro* methyltransferase activity assay was set up by adding SAM as the methyl donor, and the methylation level of tRNA was measured by dot blot with a specific antibody. The results showed that both the recombinant wild-type TRMT10A and the NoLS mutant could methylate *in vitro*-transcribed tRNA^IniMeth^ and tRNA^Gln^, but not tRNA^IniMeth^-G9C and tRNA^Gln^-G9C variants (Figure 1R), confirming the site specificity of this methyltransferase. Further experiment showed that the m^1^G modification level was lower in the NoLS mutant group than in the wild-type enzyme group at high substrate concentration (Figure 1S), consistent with previous reports that the N-terminal domain of Trm10, prokaryotic homolog of TRMT10A, helps the enzyme to recognize substrate tRNA, and tRNA genes were clustered in the nucleolus for transcription (Thompson et al., 2003; Shao et al., 2014). Taken together, these results indicate that the NoLS is critical for TRMT10A to modify its substrate tRNA in cells and also affects its enzymatic activity *in vitro*, although in a less degree.

In summary, our study demonstrates that a typical NoLS within the N-terminal domain directs TRMT10A to predominantly accumulate into the nucleolus in human cells. Altering its subcellular localization impairs its ability to catalyze tRNA m^1^G9 modification within the cells. Our finding provides evidence that the m^1^G9 of tRNAs may be modified in the nucleolus. As the nascent pre-tRNAs are transcribed and early processed in the nucleolus (Thompson et al., 2003), it is reasonable to speculate that the m^1^G9 would be one of the early tRNA modifications, which is important for proper tRNA processing.

*[Supplementary material is available at Journal of Molecular Cell Biology online. This study was supported by grants from the National Natural Science Foundation of China (82230075 to D.G.; 32270159 to J.W.), Guangdong Basic and Applied Basic Research Foundation (2023A1515012613 to J.W.), Shenzhen Science and Technology Program (JCYJ20200109142201695 and KQTD20180411143323605 to D.G.; JCYJ20220530145608018 to J.W.), and Shenzhen Key Laboratory of Systems Medicine for Inflammatory Diseases (ZDSYS20220606100803007 to J.W.). D.G. and J.W. designed the project; T.L., Z.S., and H.Y. performed most of the experiments; J.M., Z.C., J.Z., Q.Z., W.W., Y.J., X.X., L.C., H.P., and C.L. provided technical assistance; and D.G., J.W., and T.L. analyzed data and wrote the manuscript.]*

## Supplementary Material

mjaf011_Supplemental_File
