# Supplementary material for: Characterization of the nucleolar localization signal of TRMT10A and its importance for the m1G9 methylation of tRNAs in mammalian cells
Source: J Mol Cell Biol. 2025 Mar 17;17(3):mjaf011. doi: 10.1093/jmcb/mjaf011 (PMC12532308; doi:10.1093/jmcb/mjaf011)
Supplement: mjaf011_Supplemental_File [file mjaf011_supplemental_file.pdf]

Supplementary material

Characterization of the nucleolar localization signal of TRMT10A and its importance for the m^1G9 methylation of tRNAs in mammalian cells

Tianyang Luo[1,†], Zhiyuan Shi[1,†], Haibin Yang[1,†], Jiafan Miao[1], Zilong Chang[1], Jie Zou[1], Qiang Zeng[1], Wenbin Wu[1], Yanan Jiang[1], Xiaoling Xie[1], Liu Cao[1], Hong Peng[1], Chunmei Li[1], Deyin Guo[2,3,4,*], and Junyu Wu[1,*]

[1] Shenzhen Key Laboratory of Systems Medicine for Inflammatory Diseases, Centre for Infection and Immunity Study (CIIS), School of Medicine, Shenzhen Campus of Sun Yat-sen University, Shenzhen 518197, China

[2] Guangzhou National Laboratory, Guangzhou International Bio-Island, Guangzhou 510320, China

[3] MOE Key Laboratory of Tropical Disease Control, Institute of Human Virology, Department of Pathogen Biology and Biosecurity, Zhongshan School of Medicine, Sun Yat-sen University, Guangzhou 510080, China

[4] State Key Laboratory of Respiratory Disease, National Clinical Research Center for Respiratory Disease, Guangzhou Institute of Respiratory Health, the First Affiliated Hospital of Guangzhou Medical University, Guangzhou 510182, China

[†] These authors contributed equally to this work.

* Correspondence to: Deyin Guo, E-mail: guodeyin@mail.sysu.edu.cn; Junyu Wu, E-mail: wujy68@mail.sysu.edu.cn

Supplementary Materials and methods

Reagents and Antibodies

Antibodies against NPM1, TRMT10A, GFP and GAPDH were purchased from Proteintech. Antibody against 1-methylguanosine (m^1G) from Abcam. DAPI was purchased from Beyotime. Hoechst 33342 for staining nuclei of live cells was purchased from Biosharp.

Cell culture and transfection

HeLa, U251 and HEK293T cells were cultured in a Dulbecco's Modified Eagle Medium (DMEM, Gibco) containing 10% fetal bovine serum (FBS, Sigma-Aldrich), 1% Penicillin/Streptomycin (Gibco) under 5% CO_2 in a 37 ℃ incubator (Thermo Scientific). The plasmids were transfected using Lipofectamine 2000 transfection reagent (Invitrogen) according to the manufacturer’s instructions in medium without antibiotics (Opti-MEM, Gibico).

Generation of TRMT10A knock-out cell line

The pLenti-TRMT10A-sgRNA plasmid containing the sgRNA sequence targeting TRMT10A was purchased from Beyotime Institute of Biotechnology (Shanghai, China). Lentivirus was packaged by co-transfection with plasmids pLenti-TRMT10A-sgRNA, pSPAX2 and pMD2G into 293T cells. The supernatants were collected at 48 or 72 hours post-transfection and then filtered through a 0.45 μm needle filter. HeLa and 293T cells were infected with the lentivirus together with the control group for 2 days and were then selected with 2 μg/mL puromycin for 1 week. Single cell colonies were picked and the expression of TRMT10A was confirmed by Western blotting.

Plasmids construction

RNA was extracted from 293T cells using the Ultrapure RNA Kit (CWBIO). Subsequently, mRNA was reverse transcribed into cDNA using M-MLV Reverse Transcriptase (Promega). Human TRMT10A cDNA was amplified by PCR and was then cloned into the pEGFP-N1 vector. The truncation plasmids pEGFP-TRMT10A-aa1-95, pEGFP-TRMT10A-aa95-276, pEGFP-TRMT10A-aa276-339, pEGFP-TRMT10A-aa1-276 and pEGFP-TRMT10A-aa95-339 were subcloned into the same vector. The enzyme-dead mutant and NoLS mutant of TRMT10A were generated by site-directed mutagenesis PCR using PrimeSTAR GXL DNA Polymerase (Takara). All constructs were confirmed by sequencing and all the primers were listed in Table S1.

Immunofluorescence microscopy

HeLa, U251 and 293T cells were seeded into chamber slides before plasmid transfection. After 48 hours of transfection, the cells were washed with PBS and fixed in 4% paraformaldehyde for 15

minutes at room temperature. The fixed cells were permeabilized with 0.25% Triton X-100 for 10 min, blocked with 3% bovine serum albumin (BSA, ABCone) for 30 min and then incubated with the indicated primary antibodies overnight at 4 ℃. After three times wash in PBST, the cells were further incubated with Alexa Fluor 488- or 555- conjugated secondary antibodies for 1 h at room temperature and then stained with 40,6-diamidino-2-phenylindole (DAPI, Beyotime) to visualize the nuclei. Immunofluorescence images were acquired using a Zeiss LSM 900 laser confocal microscope with 100 × oil objective. Images were then processed using ZEN software (Zeiss).

Prediction of nucleolus localization sequence

NoLS motif in human TRMT10A protein was predicted using the NoD web tool (http://www.compbio.dundee.ac.uk/www-nod) (Scott et al., 2011). Then the nucleolus localization mutation of TRMT10A was constructed according to the prediction information.

Western blot and dot blot

For western blot, cells were lysed with RIPA buffer (Solarbio) on ice for 20 min. After boiled in 1x SDS-PAGE loading buffer (CWBIO) for 12 min, proteins were separated by SDS-PAGE gel electrophoresis and transfered to PVDF membrane (Millipore). The membrane was blocked in 5% skimmed milk and then incubated with appropriate primary antibodies, followed by incubation with horseradish peroxidase (HRP)-conjugated secondary antibodies (Abmart). Protein signals were visualized using Chemistar^{TM} High-sig ECL Western Blotting Substrate (Tanon).

For dot blot, small RNAs (<200 nt) were isolated using the MiPure Cell/Tissue miRNA Kit (Vazyme). In brief, the small RNAs were dropped onto the ImmobilonTM-NY+ Membrane (Millipore) in two-fold serial dilutions. After UV cross-linking, the membrane was stained with methylene blue (Sangon Biotech) and then blocked with 5% milk in TBST for 1h at room temperature. The membrane was incubated with the anti-m^{1}G antibody (Abcam) overnight at 4 ℃. After three times wash in TBST, the membrane was incubated with HRP-conjugated secondary antibody. The membrane was then visualized using Chemistar^{TM} High-sig ECL Western Blotting Substrate (Tanon).

LC-MS based RNA modification detection

LC-MS-based RNA modification detection was performed by Aksomics (Shanghai, China). In brief, total RNAs were extracted from the cells and each RNA sample was checked for integrity and quantity using agarose gel electrophoresis and a Nanodrop™ instrument. RNAs were subjected to urea-PAGE electrophoresis and the 60-90 nt small RNAs were extracted from the gel by crushing and soaking in 0.3 M sodium acetate and further purified by ethanol precipitation. The small RNAs were hydrolyzed to individual nucleosides. Then, the nucleosides were dephosphorylated using Shrimp Alkaline

Phosphatase (M0371, New England Biol). After deproteinization with a Satorius 10,000-Da MWCO spin filter, the nucleoside mixtures were analyzed using an Agilent 6460 QQQ mass spectrometer with Agilent 1260 HPLC system in multi-reaction monitoring (MRM) detection mode. The LC-MS data were acquired using Agilent Qualitative Analysis software and MRM peaks of each modified nucleoside were extracted and normalized. The Origin software was used for data visualization.

Primer extension assay

The primer extension assay was performed as previously described (Swinehart et al., 2013). In brief, the small RNAs were isolated as above. 1 μg small RNAs were reverse transcribed with 1 pmol specific biotinylated DNA primer using M-MLV Reverse Transcriptase (Promega). After reverse transcription, the products were separated by electrophoresis on a 15% urea-polyacrylamide gel (urea-PAGE) and then electroblotted onto ImmobilonTM-NY+ Membrane at 400 mA for 1 hour. Following UV cross-linking, the membrane was washed, blocked, and incubated with streptavidin-HRP. Detection was carried out using Chemistar^{TM} High-sig ECL Western Blotting Substrate. The extension primers were from published articles (Cosentino et al., 2018).

In vitro transcription of tRNAs

The DNA templates of tRNA (tRNA^{IniMeth}, tRNA^{IniMeth}-G9C, tRNA^{Gln} or tRNA^{Gln}-G9C) containing T7 RNA polymerase promoter were generated by annealing of the related primers (Supplementary Table S1) and one-step extension with 2×Accurate Taq Master Mix (AG11007, Accurate Biology). All tRNAs were *in vitro* transcribed by using T7 High Yield RNA Transcription Kit (TR101-01, Vazyme) with 1 μg template DNA. Transcription was performed at 37°C for 8 h, followed by DNase I (RNase free) treatment for 15 min. Subsequently, the transcription products were purified by TRIzol (Invitrogen Life Technologies) extraction and ethanol precipitation.

Recombinant protein expression and purification

The coding DNA of wildtype or NoLS-mutant TRMT10A was subcloned into the pET-28a-SUMO vector by homologous recombination. The constructs were transformed into *E.coli* (Rosetta (DE3)). Protein expression was induced by 0.2 mM IPTG for 20 hours at 16℃. The cells were collected and resuspended with binding buffer (20 mM Tris-Cl, pH8.5, 500 mM NaCl, 10 mM imidazole), supplemented with super nuclease and 1 mM PMSF. After lysed by French press homogenizer, the lysate was centrifuged at 25,000 rpm for 0.5 hours at 4℃. The supernatant was transferred to a gravity-based Ni2+-NTA column and washed 10 CV with washing buffer (20 mM Tris-Cl, pH 8.0, 500 mM NaCl, 20 mM imidazole). The target proteins were eluted with 300 mM imidazole and digested by SUMO protease for 1 hour at 4℃. Size-exclusion chromatography was executed on an

ÄKTA pureTM system (GE Healthcare), equipped with a Superdex TM 200pg HiLoad TM 16/600 GL column (cytiva), equilibrated with buffer (20 mM Tris-Cl, pH 7.5, 100 mM NaCl, 2 mM MgCl2). The digested elutions were loaded and separated on the column with a flow rate of 1 ml/min. The purity of recombinant proteins was detected by SDS-PAGE.

In vitro methyltransferase activity assay

In vitro m^1G9 methylation assays were performed using the *in vitro* transcribed tRNAs and the recombinant proteins as described previously (Strassler et al., 2023). The reaction mixture (20 µl) consisted of 50mM Tris–HCl, pH 7.4, 3mM MgCl2, 0.5 mM SAM, 5 µM enzyme (wildtype or NoLS-mutant TRMT10A) and 5 µg tRNA ($tRNA^{IniMeth}$, $tRNA^{IniMeth}$-G9C, $tRNA^{Gln}$ or $tRNA^{Gln}$-G9C). The reaction mixture was then incubated at 37 ℃ for 2 h. The reaction was stopped by heating at 95 ℃ for 3 min. The *in vitro* methyltransferase activity was then examined by dot blot with 3 µl of the reaction mixture as described above. The enzymatic kinetics of wild-type or NoLS-mutant TRMT10A for $tRNA^{IniMeth}$ were determined with increasing concentrations of $tRNA^{IniMeth}$ (0, 1, 1.5, 2, 4, 8, 12 µM) and 1 µM enzyme.

Supplementary References

Cosentino, C., Toivonen, S., Diaz Villamil, E., et al. (2018). Pancreatic β-cell tRNA hypomethylation and fragmentation link TRMT10A deficiency with diabetes. Nucleic Acids Res. *46*, 10302-10318.

Scott, M.S., Troshin, P.V., and Barton, G.J. (2011). NoD: a Nucleolar localization sequence detector for eukaryotic and viral proteins. BMC Bioinformatics *12*, 317.

Strassler, S.E., Bowles, I.E., Krishnamohan, A., et al. (2023). tRNA m(1)G9 modification depends on substrate-specific RNA conformational changes induced by the methyltransferase Trm10. J. Biol. Chem. *299*, 105443.

Swinehart, W.E., Henderson, J.C., and Jackman, J.E. (2013). Unexpected expansion of tRNA substrate recognition by the yeast m1G9 methyltransferase Trm10. RNA *19*, 1137-1146.

Supplementary Figures

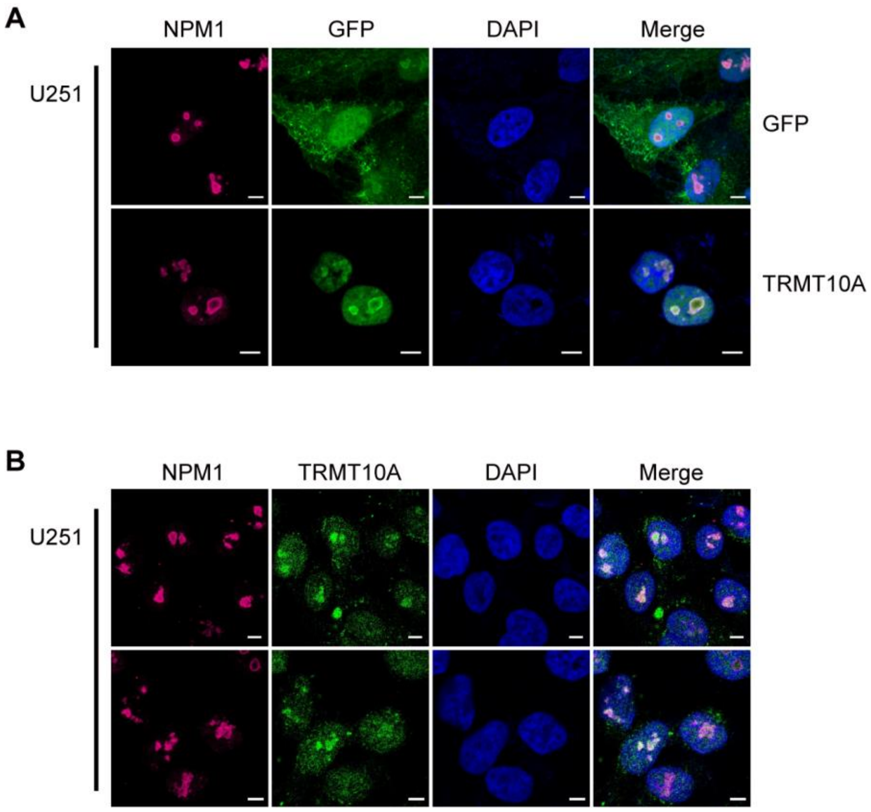

Supplementary Figure S1 TRMT10A is localized to the nucleolus.

(**A**) The subcellular distribution of GFP-tagged TRMT10A. U251 cells transfected with pEGFP-TRMT10A were stained with anti-GFP (green), anti-NPM1 (magenta) and DAPI (blue). NPM1 serves as a nucleolar marker. (**B**) Immunofluorescence staining of U251cells using anti-TRMT10A (green), anti-NPM1 (magenta) and DAPI (blue). Scale bars, 5 μm.

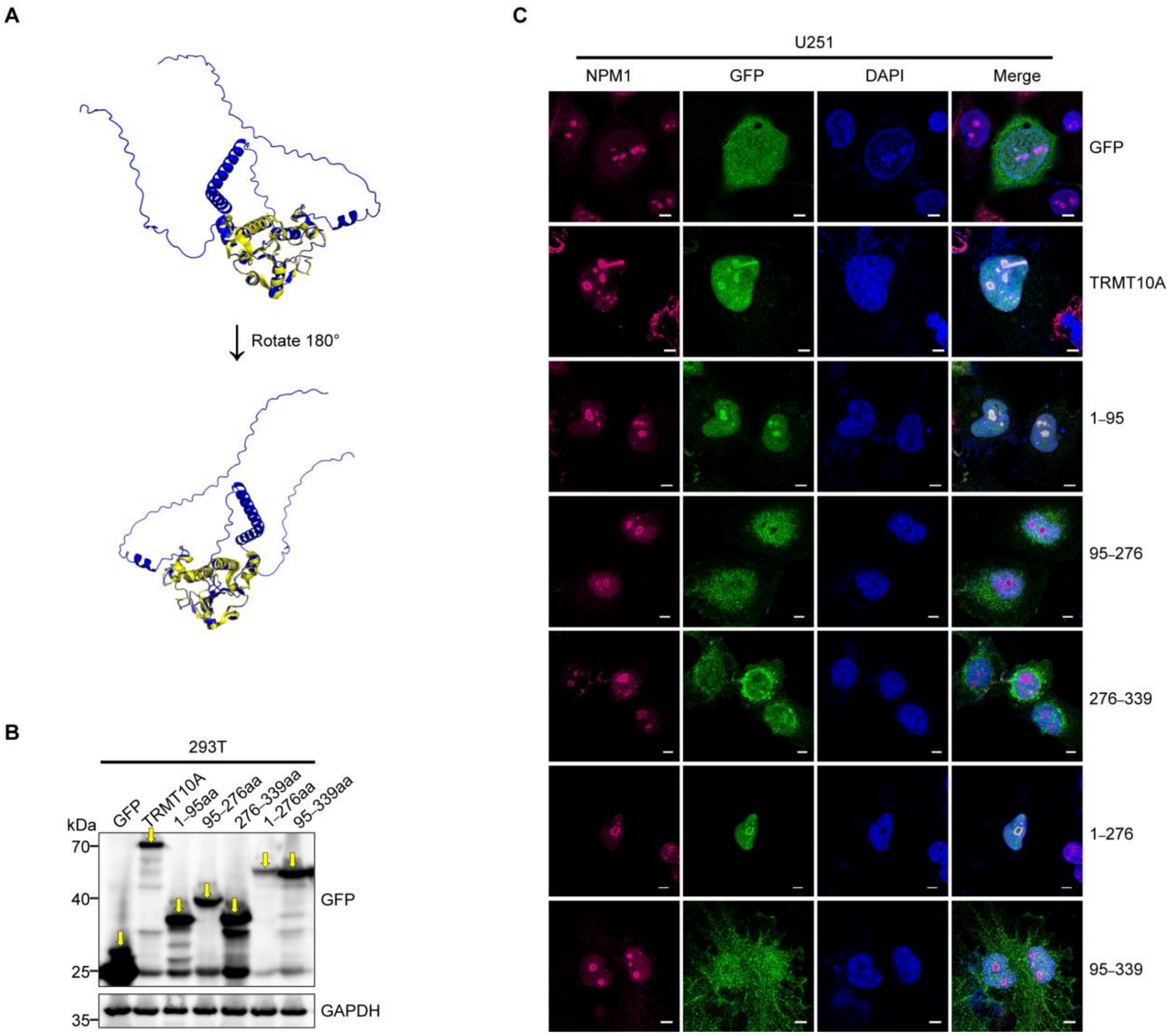

Supplementary Figure S2 The N-terminal domain is responsible for the nucleolus localization of TRMT10A.

(**A**) Overlay comparison of the predicted structure of TRMT10A (blue) with the solved crystal structure of the methyltransferase domain (PDB 4FMW, yellow) using PyMOL software. (**B**) Western blotting analysis to evaluate the protein level of GFP-tagged TRMT10A truncations. The relevant target proteins were labeled with yellow arrows. (**C**) The subcellular distribution of GFP-tagged TRMT10A truncations in U251 cells. U251 cells transfected with related plasmids were stained with anti-GFP (green), anti-NPM1 (magenta) and DAPI (blue). Scale bars, 5 μm.

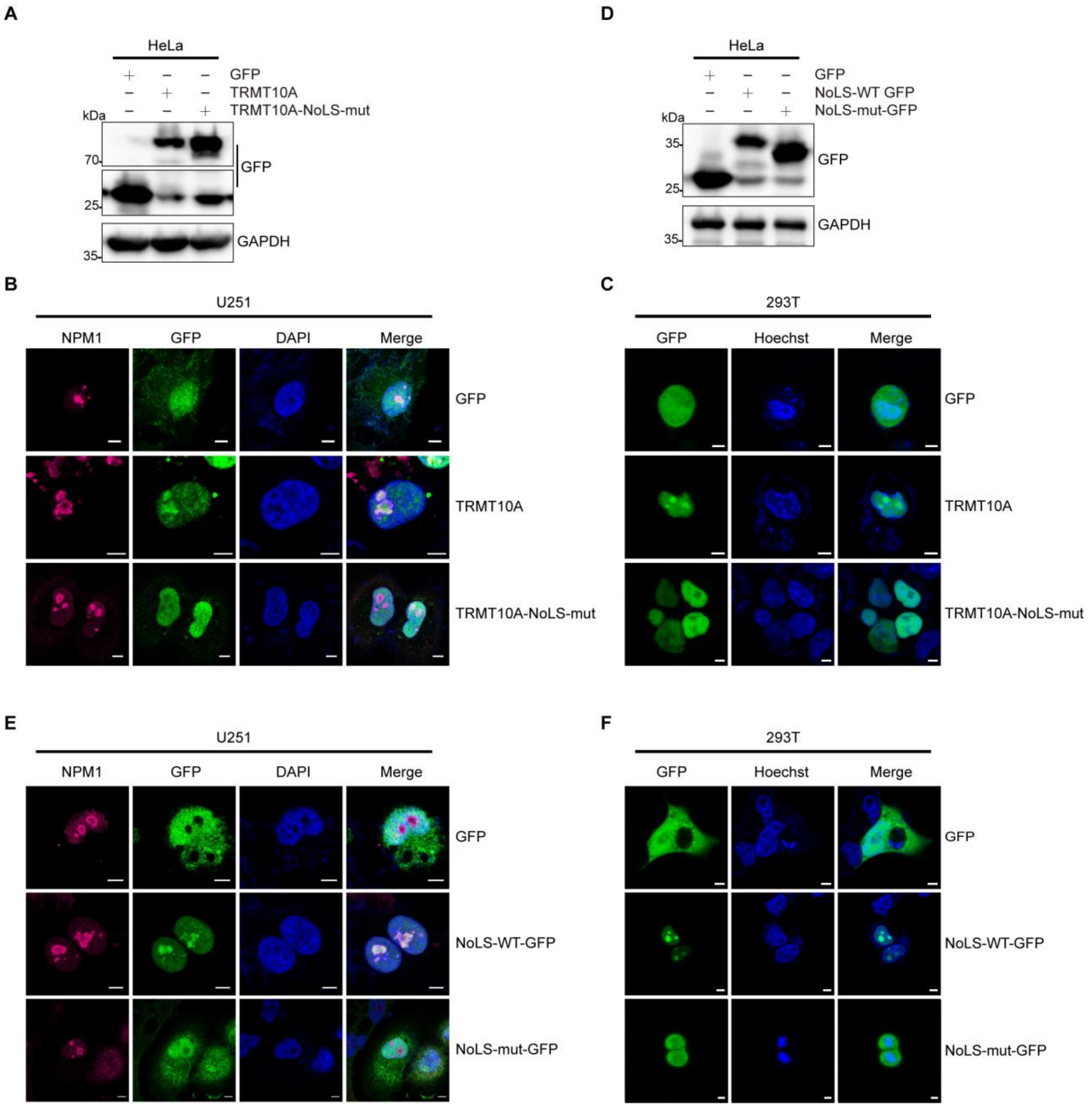

Supplementary Figure S3 The verification of TRMT10A-NoLS in U251 and 293T cells.

(**A**) The expression of GFP-tagged TRMT10A (wild-type or NoLS mutant) were detected by Western blot. (**B** and **C**) The NoLS mutation blocked the nucleolus localization of TRMT10A. The subcellular localizations of TRMT10A and TRMT10A-NoLS-mut were visualized in U251 cells (**B**) and in 293T cells (**C**). (**D**) The expression of the wild-type or mutant NoLS-GFP fusion protein were assessed by Western blot. (**E** and **F**) The NoLS of TRMT10A is sufficient to locate protein to the nucleolus. The NoLS of TRMT10A and its mutated sequence were fused with GFP, and the subcellular localization of these fusion proteins were visualized in U251 cells (**E**) and in 293T cells (**F**). Scale bars, 5 μm.

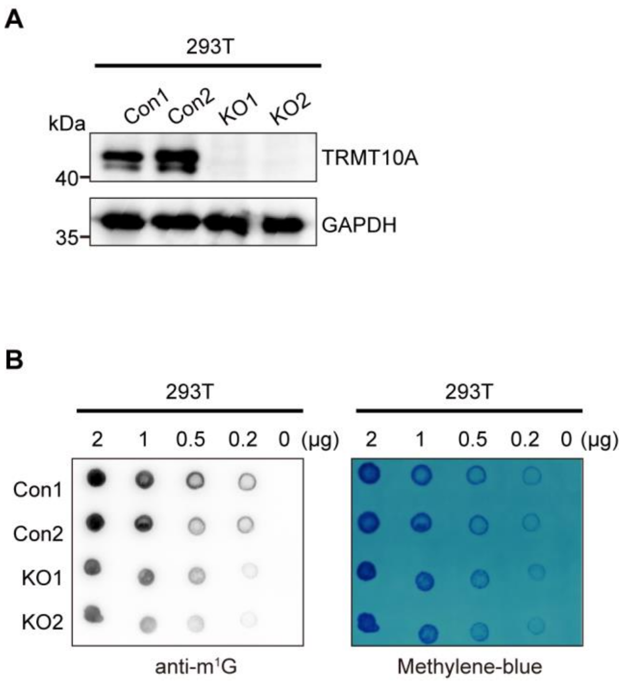

Supplementary Figure S4 TRMT10A is the major methyltransferase for m^{1}G9 methylation on tRNAs.

(**A**) Western blotting analysis to confirm the knockout efficiency of TRMT10A in 293T cells. Two control cell lines and two TRMT10A knockout cell lines were used. GAPDH was used as loading control. (**B**) Dot blot analysis to detect m^{1}G modification levels of small RNAs (<200 nt) isolated from TRMT10A knockout and wild-type 293T cells. The methylene blue staining was the loading control.

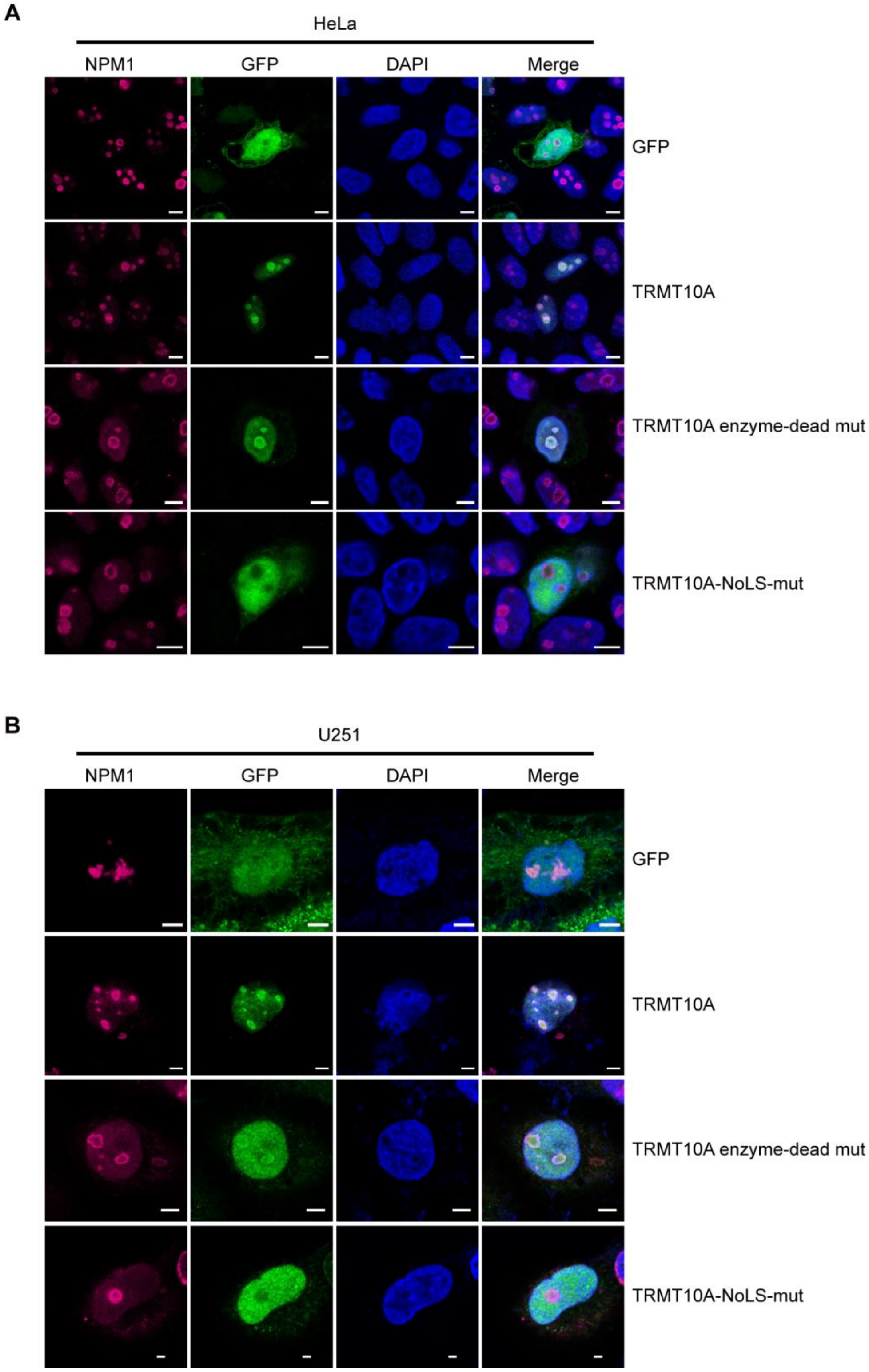

Supplementary Figure S5 The enzyme-dead mutation in TRMT10A did not affect its nucleolar localization.

HeLa (**A**) and U251 (**B**) cells transfected with related plasmids were stained with anti-GFP (green), anti-NPM1 (magenta) and DAPI (blue). Scale bars, 5 μm.

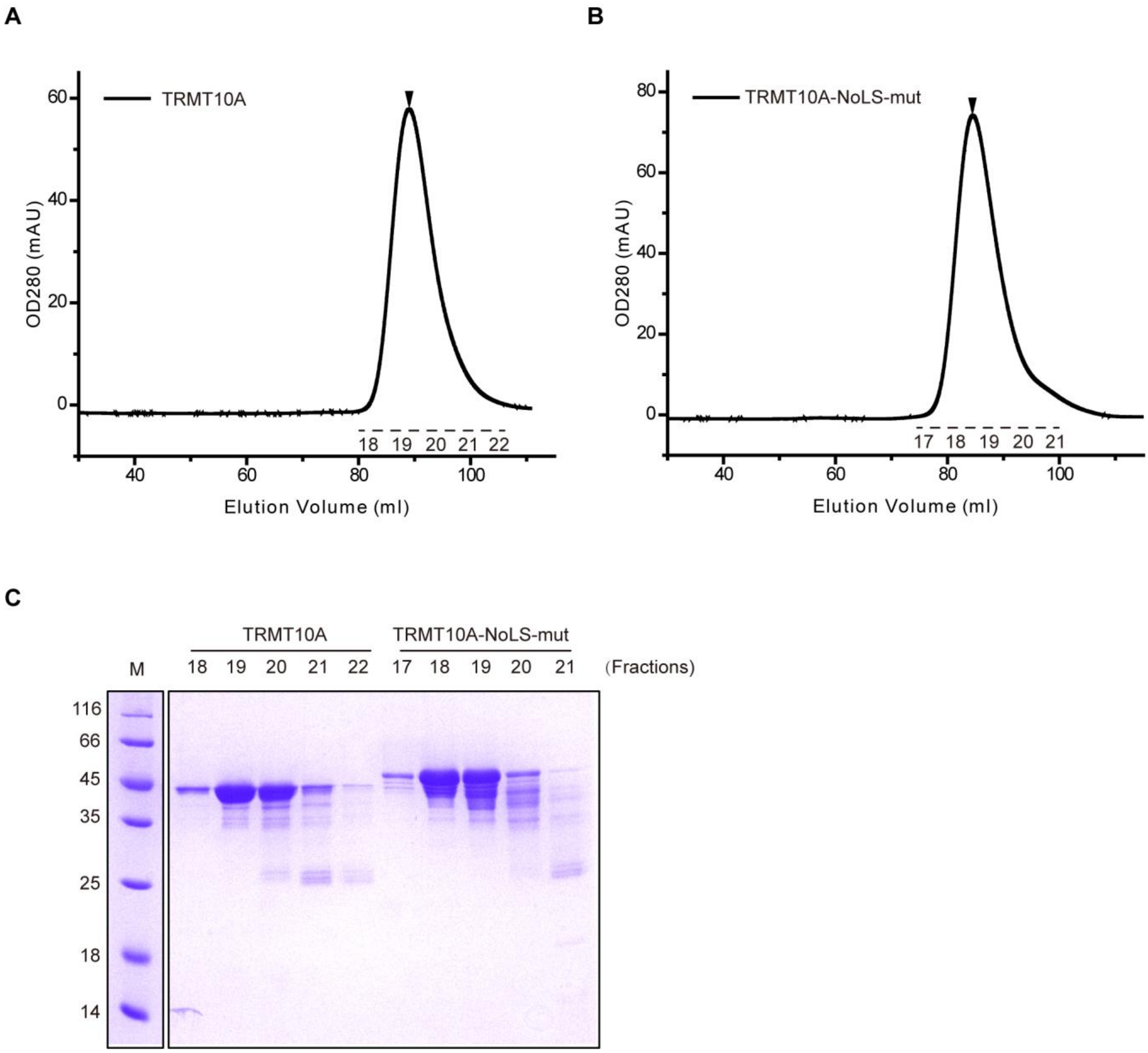

Supplementary Figure S6 Biochemical characterization of TRMT10A purification.

(**A** and **B**) Size-exclusion chromatogram of wildtype and NoLS-mutant TRMT10A using ÄKTApurifier, the x-axis indicates the elution volume, the y-axis indicates the UV absorption, the black arrow shows the target peak with the fraction tube number labelled under UV curve, the mutated amino acids are indicated in method part. (**C**) SDS-PAGE analysis of TRMT10A purifying fractions, stained by Coomassie brilliant blue G-250. The molecular mass of target bands is indicated by comparing standard molecular markers in the first lane, the unit is kDa.

Supplementary Table

Supplementary Table S1 Primers and probes used in this study.

**Primer name**	**Sequence (5′-> 3′)**
hTRMT10A-clone-pEGFP-N1-F1	CGGCTAGCGCCACCATGTCATCTGAAATGTTG
hTRMT10A-clone-pEGFP-N1-R339	CGGGATCCCCGTGTGGCAGAGAGTT
hTRMT10A-clone-pEGFP-N1-F95	CGGCTAGCGCCACCATGGATGTTGTTCATAGCAC
hTRMT10A-clone-pEGFP-N1-R95	CGGGATCCCCAACATCTCTTCGAACACG
hTRMT10A-clone-pEGFP-N1-F276	CGGCTAGCGCCACCATGAAAGGAGCTGTTCCCACAG
hTRMT10A-clone-pEGFP-N1-R276	CGGGATCCCCTTTCCGTTGGGGCAAG
hTRMT10A-enzyme-mut-F	CTATGTGATTAGAGGATTAGCAGCTCACAACCATCACA AGGGAC
hTRMT10A-enzyme-mut-R	CTTGTGATGGTTGTGAGCTGCTAATCCTCTAATCACAT AGGCCTTTGATTC
hTRMT10A-NoLS-mut-F-RK-A	CGGGAACTCGCCGCACAAGCGGCAGCAGAAGCAGCC GCGGCGGCAGCATTAGAGCGACAATGTC
hTRMT10A-NoLS-mut-R-RK-A	GTCGCTCTAATGCTGCCGCCGCGGCTGCTTCTGCTGCC GCTTGTGCGGCGAGTTCCCGTTGCTC
TRMT10A-NoLS sequence-F	CGGCTAGCGCCACCATGTGTGAACCAATATC
TRMT10A-NoLS sequence-R	CGGGATCCCCTGAGTTTGGTTCCAT
$tRNA^{Gln}_{(UUG/CUG)}$-probe	ACTCGGATCGCTGGATTCA
$tRNA^{IniMeth}_{(CAU)}$-probe	TCCATCGACCTCTGGGTTAT
htRNAiMet-G9-F	GACTTCATCGATAATACGACTCACTATAGAGCAGAGTG GCGCAGCGGAAGCGTGCTGGGCCCA
htRNAiMet-G9C-F	GACTTCATCGATAATACGACTCACTATAGAGCAGAGTC GCGCAGCGGAAGCGTGCTGGGCCCA
htRNAiMet-G9-R	TAGCAGAGGATGGTTTCGATCCATCGACCTCTGGGTTA TGGGCCCAGCACGCTTCCGCTGCG
htRNAGln-G9-F	GACTTCATCGATAATACGACTCACTATAGGGTTCCATG GTGTAATGGTTAGCACTCTGGACTCTGA
htRNAGln-G9C-F	GACTTCATCGATAATACGACTCACTATAGGGTTCCATC GTGTAATGGTTAGCACTCTGGACTCTGA
htRNAGln-G9-R	AGGTTCCACCGAGATTTGAACTCGGATCGCTGGATTC AGAGTCCAGAGTGCTAACCATTACA
